# Protective Effects of Fermented Soybeans (*Cheonggukjang*) on Dextran Sodium Sulfate (DSS)-Induced Colitis in a Mouse Model

**DOI:** 10.3390/foods11060776

**Published:** 2022-03-08

**Authors:** Hyeon-Ji Lim, Ha-Rim Kim, Su-Ji Jeong, Hee-Jong Yang, Myeong Seon Ryu, Do-Youn Jeong, Seon-Young Kim, Chan-Hun Jung

**Affiliations:** 1 Jeonju AgroBio-Materials Institute, Wonjangdong-gil 111-27, Jeonju 54810, Korea; lhj0923@jami.re.kr (H.-J.L.); poshrim@jami.re.kr (H.-R.K.); 2 Microbial Institute for Fermentation Industry, Sunchang 56048, Korea; yo217@naver.com (S.-J.J.); godfiltss@naver.com (H.-J.Y.); rms6223@naver.com (M.S.R.); jdy2534@korea.kr (D.-Y.J.)

**Keywords:** inflammatory bowel disease, *Cheonggukjang*, dextran sulfate sodium (DSS)-induced colitis, protective effect, functional food

## Abstract

Inflammatory bowel disease (IBD) is a chronic inflammatory disease, and the incidence of IBD is increasing every year owing to changes in dietary structure. Although the exact pathogenesis of IBD is still unclear, recent evidence suggests that gut dysbiosis is closely associated with IBD pathogenesis. *Cheonggukjang* is a traditional Korean fermented soybean paste produced using traditional and industrial methods, and contains probiotics, which affect the gut microbiota composition. However, the protective effect of *Cheonggukjang* against IBD is unknown. In this study, we investigated the bacterial community structure of traditional and commercial *Cheonggukjang* samples, as well as the protective effect of *Cheonggukjang* on a dextran sulfate sodium (DSS)-induced colitis mouse model. Traditional and commercial *Cheonggukjang* were found to contain various type of useful probiotics in their bacterial community structure. *Cheonggukjang* reduced the progression of DSS-induced symptoms, such as body weight loss, colonic shortening, disease activity index, and histological changes. Further, *Cheonggukjang* improved the intestinal epithelial barrier integrity on DSS-induced colitis mice. In addition, *Cheonggukjang* suppressed the expression of proinflammatory cytokines and inflammatory mediators through the inactivation of NF-κB and MAPK signaling pathways. These results indicate that *Cheonggukjang* exerts protective effects against DSS-induced colitis, suggesting its possible application as a functional food for improving inflammatory diseases.

## 1. Introduction

Inflammatory bowel disease (IBD) is a chronic inflammatory disease of the intestine, and its incidence is increasing every year owing to changes in the structure of diets [1,2,3]. Patients with IBD are known to have a lower quality of life than healthy individuals due to abdominal cramping, diarrhea, bloody diarrhea, fever, fatigue, symptoms of weight loss, and a higher risk of colitis-associated colorectal cancer [4]. Although many studies have shown that multiple factors, including genetic, microbial, environmental, and immune-mediated factors, are associated with IBD, its exact pathogenesis is complex and still unclear [2,5]. However, recent evidence suggests that gut dysbiosis is associated with IBD pathogenesis [6]. Under normal conditions, the mucosal immune system is precisely regulated; however, disruption of normal mucosal immunity to commensal microbiota results in chronic intestinal inflammation, and, consequently, IBD [7,8]. Proinflammatory cytokines, such as *IL-1β*, *IL-6*, and *TNF-α*, which are activated by the nuclear factor-κB (NF-κB) and mitogen-activated protein kinase (MAPK) signaling pathways, play a crucial role in the colonic mucosal immune response in intestinal inflammation in patients with IBD [9,10,11].

*Cheonggukjang* is a traditional Korean fermented paste made by short-term fermentation of soybeans [12]. Various enzymes and physiologically active substances, such as dietary fiber, phosphatide, isoflavone, flavonoids, phenolic acids, saponins, trypsin inhibitors, and poly glutamic acid, are produced during *Cheonggukjang* fermentation [13]. These components show various biological activities, such as antioxidant, anti-atherosclerosis, anti-obesity, anti-diabetes, blood pressure-lowering, and osteoporosis prevention properties [13,14,15]. Additionally, probiotic strains, *Bacillus* and *Lactobacillus*, which were the dominant microbes at the genus level, have been reported in *Cheonggukjang* [16]. These probiotics are known to exert beneficial effects, including immune modulation, modulation of gut microbiota, displacement of pathogens, and production of bioactive compounds in the gastrointestinal tract of the host [17,18]. However, the protective effect of *Cheonggukjang* against IBD is unknown.

*Cheonggukjang* is typically produced using traditional or commercial methods, and its physicochemical and functional properties differ depending on the manufacturing method, soybean variety, microorganisms, and fermentation time [19]. Nowadays, consumers are highly interested in traditionally made *Cheonggukjang* products due to their consistent outstanding sensory quality. However, while traditionally made *Cheonggukjang* fermented with various regional microorganisms has better taste and aroma than commercial products fermented using certain strains, the functional difference is unknown [20]. Therefore, this study aimed to evaluate the protective effect of *Cheonggukjang* in a dextran sulfate sodium (DSS)-induced colitis mouse model, and the functional differences between traditional and commercial *Cheonggukjang*.

## 2. Materials and Methods

### 2.1. Antibodies

In this study, the following antibodies were used: anti-iNOS, anti-COX-2, anti-p-p38, anti-p38, anti-p-ERK, anti-p-JNK, anti-p-p65, anti-p65, anti-occludin, and anti-β-actin from Cell Signaling Technology (Danvers, MA, USA); anti-ERK and anti-JNK from Santa Cruz Biotechnology (Dallas, TX, USA); anti-ZO-1 from Abcam (Cambridge, UK).

### 2.2. Preparation of Cheonggukjang Samples

For this study, four different types of *Cheonggukjang* were obtained from the Microbial Institute for Fermentation Industry (Sunchang-gun, Jeollabuk-do, Korea). Moisture content and sample information of the *Cheonggukjang* samples were as follows: (1) S1 (60.94%, Sunchang-gun, Jeollabuk-do, Korea), (2) S2 (53.15%, Kangjin-gun, Jeollabuk-do, Korea), (3) S3 (48.45%, Paju-si, Gyeonggi-do, Korea), and (4) S4 (51.57%, Sunchang-gun, Jeollabuk-do, Korea). The *Cheonggukjang* samples S1–S3 were traditionally made, whereas the *Cheonggukjang* sample S4 was a commercial brand sample. The samples were dissolved in distilled water at 500 mg/kg, and then stored at −20 °C before oral administration to mice.

### 2.3. Bacterial Community Analysis of Cheonggukjang by Next-Generation Sequencing (NGS)

Bacterial community analysis of *Cheonggukjang* was performed using an NGS, as described previously [21]. Briefly, the total DNA from the collected *Cheonggukjang* samples was extracted by DNeasy PowerSoil Kit (Qiagen, Hilden, Germany), and amplified with V3-V4 regions of 16S rRNA gene targeting primers. Libraries of the PCR amplicon were prepared by Nextera XT DNA Library Prep Kit (Illunina, San Diego, CA, USA), and sequencing was performed using 300 bp paired-end reads on the Illumina Miseq platform at the Microbial Institute for Fermentation Industry (Sunchang, South Korea). Obtained raw fastq data were analyzed using Mothur package v. 1.36. Chimeric, low-quality, and non-bacterial reads were removed, and the remaining sequences were grouped into single operational taxonomic units (OTUs) against the SILVA bacterial database v. 12350, and all reads within 97% similarity were clustered by a single OTUs sequence. Sequences were taxonomically classified at different levels (phylum, class, order, family, genus, and species). The bacterial clustering of each sample collected from different regions was performed by principal component analysis using the R package. The α-diversity indices, such as Chao and Shannon, were calculated by the Mothur program.

### 2.4. Experimental Animals

Specific pathogen-free (SPF)-grade BALB/c mice (male, 5-week-old, n = 35) were purchased from Damool Science (Daejeon, Korea), and acclimated for a week. The mice were housed in a room maintained on a 12 h light/dark cycle at 22 ± 2 °C and a relative humidity of 55 ± 5%. All animals were cared for according to the guidelines of the Animal Care Committee of Jeonju AgroBio-Materials Institute (Jeonju, Korea). All experimental procedures were approved by the Animal Care Committee of Jeonju AgroBio-Materials Institute (JAMI IACUC 2021001, Jeonju, Korea).

### 2.5. DSS-Induced Colitis and Cheonggukjang Treatment

The animals were divided into seven groups (five mice/group) according to the treatment: NOR group (normal control), DSS group [5% DSS (MP Biomedicals, Irvine, CA, USA)], PC group [positive control; 5% DSS + 50 mg/kg/day of 5-aminosalicylic acid (5-ASA; Sigma-Aldrich, St. Louis, MO, USA)], S1 group (5% DSS + 500 mg/kg/day of S1 sample), S2 group (5% DSS + 500 mg/kg/day of S2 sample), S3 group (5% DSS + 500 mg/kg/day of S3 sample), and S4 group (5% DSS + 500 mg/kg/day of S4 sample). The NOR and DSS groups were administered distilled water. *Cheonggukjang* samples (S1–S4) were orally administered at a dose of 500 mg/kg once a day for 15 days. To induce colitis, mice were administered 5% DSS in drinking water for seven days, and then sacrificed after one day.

### 2.6. Disease Activity Index (DAI)

DAI was evaluated, as described previously [22]. Briefly, the severity of colonic inflammation was assessed by summing the scores for weight loss, stool viscosity, and stool bleeding status, as shown in Table 1.

### 2.7. ELISA

Serum levels of *TNF-α*, *IL-6*, and *IL-1β* were determined using ELISA kits (R&D Systems R&D Systems, Minneapolis, MN, USA), in accordance with the manufacturer’s protocol.

### 2.8. Quantitative Real-Time PCR (qRT-PCR)

Mouse colon tissues were homogenized using ice-cold TRIzol reagent (MRC, Cincinnati, OH, USA). cDNA was synthesized by reverse transcription of 1 μg of RNA samples using a cDNA Synthesis Kit (Bio-Fact, Daejeon, Korea). The relative mRNA levels were calculated using the comparative Ct method. Β-actin was used as the reference gene. The primer sequences are listed in Table 2.

### 2.9. Western Blot Assay

Colon tissues were homogenized in a lysis buffer (Thermo Scientific, Rockford, MD, USA) containing a protease inhibitor cocktail (GenDEPOT, Katy, TX, USA). The total protein samples (25 μg per lane) were separated by SDS-PAGE, and electroblotted onto a PVDF membranes (Merck Millipore, Billerica, MA, USA). Membranes were analyzed using the specified antibodies using ECL kit (GE Healthcare, Buckinghamshire, UK), and the images were captured using an Amersham Imager 600 (GE Healthcare).

### 2.10. Histological Analysis

Colon tissues were fixed with 10% formalin and embedded in paraffin. Tissue sections (4 μm thick) were stained with hematoxylin and eosin (H&E) and Alcian blue. Images were analyzed using a microscope (Olympus, Tokyo, Japan). Colon tissue damage was scored, as described previously [23].

### 2.11. IHC Staining

Paraffin sections (4 μm thick) were deparaffinized with xylene three times for 7 min, and rehydrated using ethanol and water. Peroxidase activity was blocked using 0.3% H_2_O_2_ for 15 min. Antigen retrieval was performed with 0.01 M citrate buffer (pH 6.0) in a microwave for 15 min. The tissue sections were pre-blocked with 4% bovine serum albumin for 30 min, and then incubated overnight at 4 °C with antibodies, followed by an anti-Rabbit Envision plus polymer kit (Dako, Glostrup, Denmark). The sections were stained with hematoxylin. Images were analyzed using a microscope (Olympus).

### 2.12. Statistical Analysis

Statistical analyses were performed using Tukey’s post-hoc tests with GraphPad Prism (version 5.0; GraphPad Software, Inc., San Diego, CA, USA). Data are presented as the mean ± standard deviation (SD). For all experiments, a *p*-value < 0.05 was considered statistically significant.

## 3. Results and Discussion

### 3.1. Bacterial Community Structure in Cheonggukjang Samples

**Figure 1:**
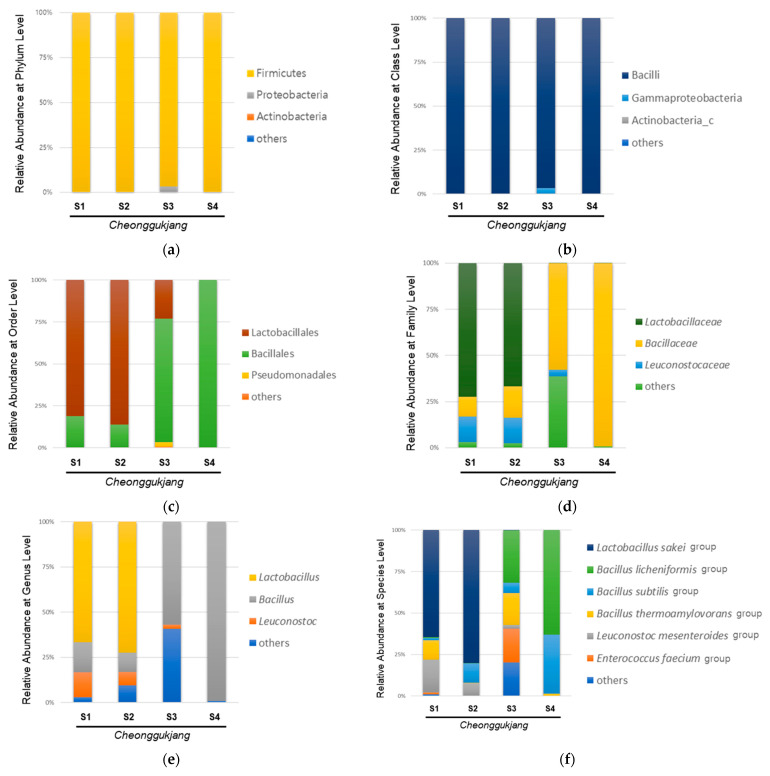
Microbial composition in the cheonggukjang samples (S1–S4): Relative abundance (%) at (**a**) the phylum level; (**b**) class level; (**c**) order level; (**d**) family level; (**e**) genus level; (**f**) species level.

The gut microbiota plays an essential role in the progression of intestinal inflammation in IBD, and it is known that IBD is associated with an imbalance of intestinal bacteria [24]. Probiotic strains can be used in the treatment and prevention of IBD in animal models of colitis, although the exact mechanism is unknown [18]. Various biological and pharmacological properties of *Cheonggukjang* have been demonstrated in animal models [13,14,15]. Furthermore, probiotics in cheonggukjang have been reported to relieve gut dysbiosis, which is closely associated with IBD [6]. However, several studies reported that traditionally made *Cheonggukjang* showed better bioactivity, such as glucose dysregulation, memory impairment, and immunity, compared to commercial *Cheonggukjang* [20]. These differences have been reported to be associated with bacteria-driven changes in the fermentation process [15]. Therefore, we performed 16S rRNA sequencing using NGS to analyze the bacterial community of traditionally made *Cheonggukjang* samples from different regions (S1, Sunchang-gun, Jeollabuk-do, South Korea; S2, Gangjin-gun, Jeollabuk-do, South Korea; S3, Paju-si, Gyeonggi-do, South Korea) and a commercial *Cheonggukjang* brand sample (S4). The relative abundances of different bacteria in the *Cheonggukjang* samples (S1–S4) at the phylum and class levels are shown in Figure 1a,b.

All *Cheonggukjang* samples (S1–S4) were dominated by *Firmicutes* and *Bacilli* at the phylum and class levels, respectively. As shown in Figure 1c, the commercial (S4) and traditionally made *Cheonggukjang* (S1–S3) samples showed different bacterial community structure at the order level. *Lactobacillales* showed the highest abundance in the traditionally made *Cheonggukjang* samples (S1, 81.1%; S2, 86.2%), except for S3 (*Lactobacillales*, 23.1%; *Bacillales*, 73.5%), whereas, *Bacillales* showed the highest abundance in the commercial cheonggukjang sample (S4) (99.9%). As shown in Figure 1d,e, the traditionally made *Cheonggukjang* samples S1 and S2 were dominated by *Lactobacillaceae* (S1, 66.6%; S2, 72.3%) and *Lactobacillus* (S1, 66.5%; S2, 72.3%) at the family and genus levels, respectively. The traditionally made S3 and commercial *Cheonggukjang* (S4) samples were dominated by *Bacillaceae* (S3, 57.7%; S4, 99.2%) and *Bacillus* (S3, 56.9%; S4, 99.1%) at the family and genus levels, respectively. At the species level, the traditionally made *Cheonggukjang* samples S1 and S2 were dominated by the *Lactobacillus sakei* group (S1, 44.8%; S2, 72.1%), while the traditionally made S3 and commercial *Cheonggukjang* (S4) samples were dominated by the *Bacillus licheniformis* group (S3, 30.0%; S4, 62.0%). Among the traditionally made *Cheonggukjang*, the *Enterococous faecium* group was observed to be higher in S3 (Gyeonggi-do, Korea) than S1 and S2 (Jeollabuk-do, Korea). Since *Cheonggukjang* is fermented by various microorganisms present in the manufacturing environment [16], the difference of bacterial community in traditionally made *Cheonggukjang* samples could be due to region-specific microorganisms. These results suggest that the bacterial community of *Cheonggukjang* may differ depending on the manufacturing method used and region.

### 3.2. Cheonggukjang Attenuates the Progression of DSS-Induced Colitis

**Figure 2:**
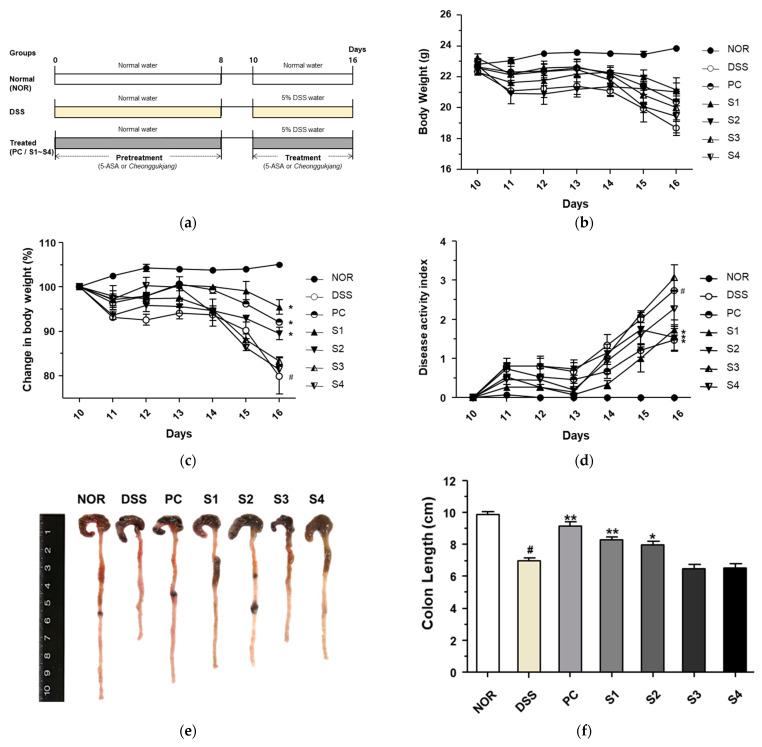
Protective effect of *Cheonggukjang* on the progression of dextran sulfate sodium (DSS)-induced colitis. (**a**) Schematic representation for animal experiments; (**b**) Body weight (g); (**c**) Change in body weight (%); (**d**) Disease activity index; (**e**) Representative images of colon tissue in each group; (**f**) Colon length (cm). Values are presented as the mean ± standard deviation (SD) (n = 5); #, *p* < 0.005 versus normal group; **, *p* < 0.005; *, *p* < 0.05 versus DSS group.

To evaluate the protective effect of *cheonggukjang* against DSS-induced colitis, we designed an animal experiment, as shown in Figure 2a. To evaluate the disease progression in DSS-induced colitis, we first measured body weight lost. As shown in Figure 2b,c, mice in the DSS-treated group had a considerable body weight loss compared to the mice in the normal group (NOR), and the reduced body weight after treatment with DSS was considerable restored by administration of 5-ASA (PC) and *Cheonggukjang* samples S1 and S2. Symptoms of DSS-induced colitis were evaluated using the DAI, which is based on body weight loss, rectal bleeding, and stool consistency (Table 1). As shown in Figure 2d, the DAI of the DSS group was considerably higher than that of the NOR group. However, treatment with PC and *Cheonggukjang* samples S1 and S2 considerable ameliorated the DAI compared to the DSS only group. Next, to confirm the protective effect of *Cheonggukjang* samples against the progression of DSS-induced colitis, we evaluated the reduction in colon length, an indicator of the severity of intestinal inflammation in DSS-induced colitis. As shown in Figure 2e,f, colon length was considerable shorter in the DSS group than in the NOR group. However, this phenomenon was considerable alleviated in the PC and *Cheonggukjang* (S1, S2) treated groups. According to an analysis of bacterial community, the S1 and S2 samples were dominated by *Lactobacillus* and the S3 and S4 samples were dominated by *Bacillus* at Genus level. *Lactobacillus* and *Bacillus* strains are widely used as probiotics, and are known to benefit the gut environment [17,18]. Therefore, we found that *Lactobacillus* may have a more protective effect than *Bacillus* in DSS-induced colitis. These findings suggest that *Cheonggukjang* containing probiotics exerts a protective effect on the progression of DSS-induced colitis. We also investigated whether cheonggukjang could affect the gut environment, but did not find any significant differences of bacterial community between the groups (data not shown).

### 3.3. Cheonggukjang Improves Histological Changes on DSS-Induced Colitis

**Figure 3:**
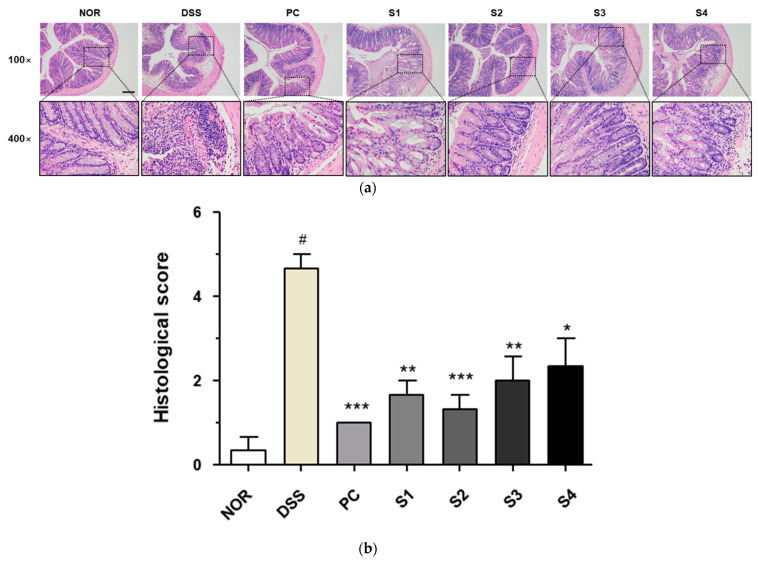
Histological changes in colorectal tissues of DSS-induced colitis mice: (**a**) Representative H&E images of colon tissue. Scale bar; 100 μm; (**b**) Histological score. Values are presented as the mean ± SD (n = 5); #, *p* < 0.005 versus normal group; ***, *p* < 0.001; **, *p* < 0.005; *, *p* < 0.05 versus DSS group.

Under normal conditions, the colorectal tissue consists of the epithelium, crypt structure, mucosa layer, and mucosa substratum. However, colonic inflammation induced by DSS induces histological changes, such as irregular surface epithelium, depleted goblet cells, distorted and shallow crypt structures, and increased inflammatory cell infiltration [25]. To evaluate histological changes, we performed H&E staining and determined the histological score, as previously described [23]. As shown in Figure 3a, the DSS group showed depleted goblet cells and increased inflammatory cell infiltration in colon tissue. These histological changes were considerably ameliorated in the PC- and *Cheonggukjang* (S1–S4)-treated groups. As shown in Figure 3b, the increased histological score in the DSS group was considerable reduced in the PC- and *Cheonggukjang*-treated groups. These results suggest that *Cheonggukjang* improves histological changes in mice with DSS-induced colitis.

### 3.4. Cheonggukjang Reduces the Expression of Proinflammatory Cytokines on DSS-Induced Colitis

**Figure 4:**
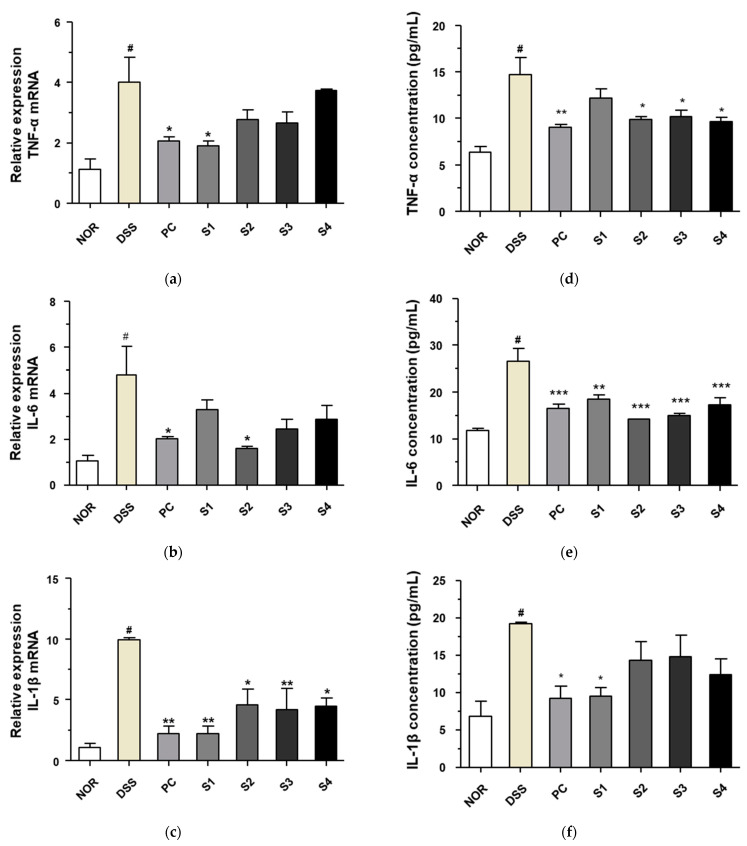
Inhibitory effect of *Cheonggukjang* on the mRNA and protein secretion of proinflammatory cytokines in DSS-induced colitis mice: mRNA levels of (**a**) *TNF-α*, (**b**) *IL-6*, and (**c**) *IL-1β* in colonic tissues; Protein levels of (**d**) *TNF-α*, (**e**) *IL-6*, and (**f**) *IL-1β* in the serum. Values are presented as the mean ± SD (n = 5); #, *p* < 0.005 versus normal group; ***, *p* < 0.001; **, *p* < 0.005; *, *p* < 0.05 versus DSS group.

DSS-induced colitis is associated with the induction of proinflammatory cytokines, such as *TNF-α*, *IL-6*, and *IL-1β* [26], and its expression is modulated by probiotics [27]. Thus, we investigated the expression of these markers to evaluate the effect of *Cheonggukjang* samples (S1–S4) containing probiotics on inflammation in DSS-induced mice. As shown in Figure 4a–c, the DSS group showed increased mRNA levels of *TNF-α*, *IL-6*, and *IL-1β* in colonic tissue. These increased mRNA levels were reduced significantly in the PC- and *Cheonggukjang-*(S1–S4) treated groups. Next, to confirm these effects, we evaluated the secretion of *TNF-α*, *IL-6*, and *IL-1β* in the blood. The secretion of *TNF-α*, *IL-6*, and *IL-1β* in the blood was increased in the DSS alone group, compared to those in the control group, as shown in Figure 4d–f. This increased secretion was significantly reduced in the PC- and *Cheonggukjang* (S1–S4)-treated groups, similar to the findings at the mRNA level. These results suggest that *Cheonggukjang* inhibits the mRNA and protein secretion of proinflammatory cytokines in mice with DSS-induced colitis.

### 3.5. Cheonggukjang Suppresses Activation of NF-κB and MAPKs Signaling Pathways on DSS-Induced Colitis

**Figure 5:**
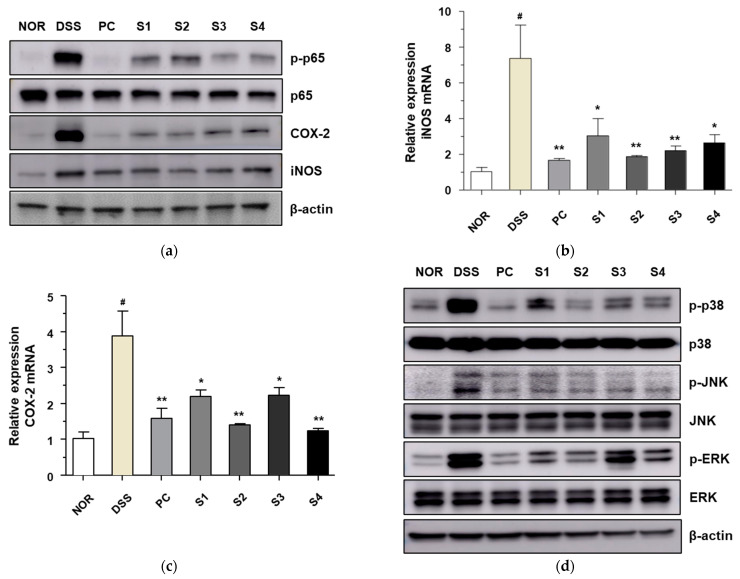
Inhibitory effect of *Cheonggukjang* on NF-κB and MAPKs signaling pathway in DSS-induced colitis mice: (**a**) The protein levels of phosphorylated p65, p65, COX-2, and iNOS were determined via western blot assay; mRNA levels of (**b**) iNOS and (**c**) COX-2 were analyzed by quantitative real-time PCR; (**d**) The protein levels of total and phosphorylated p38, JNK, and ERK were determined via western blot assay. Values are presented as the mean ± SD (n = 5); #, *p* < 0.005 versus normal group; **, *p* < 0.005; *, *p* < 0.05 versus DSS group.

NF-κB and MAPK are important signaling pathways that play a role in inducing the inflammatory response in DSS-induced colitis models [28]. Therefore, we measured the expression of these signaling molecules to evaluate the inhibitory activity of *Cheonggukjang* against DSS-induced colitis. As shown in Figure 5a, the level of phosphorylated NF-κB p65 was significantly increased in the DSS alone group compared to that in the control group. DSS-induced phosphorylation of NF-κB p65 was significantly decreased upon treatment with PC and *Cheonggukjang* (S1–S4). Levels of inflammatory enzymes, such as COX-2 and iNOS, are controlled by the proinflammatory transcription factor NF-κB p65 [29]. Thus, we evaluated the protein and mRNA levels of COX-2 and iNOS. The protein levels of COX-2 and iNOS were increased in the DSS alone group compared to those in the control group, similar to the expression of NF-κB p65. DSS-induced protein levels of COX-2 and iNOS decreased significantly upon treatment with PC and *Cheonggukjang* (S1–S4). We also confirmed the mRNA levels of COX-2 and iNOS after treatment with PC and *Cheonggukjang* (S1–S4) (Figure 5b,c). Next, we evaluated the effect of *Cheonggukjang* samples (S1–S4) on the MAPK signaling pathways. As shown in Figure 5d, the levels of phosphorylated p38, JNK, and ERK were significantly increased in the DSS alone group, compared to those in the control group. DSS-induced phosphorylation of p38, JNK, and ERK decreased significantly upon treatment with PC and *Cheonggukjang* (S1–S4). These results suggest that *Cheonggukjang* can attenuate DSS-induced colonic inflammation by modulating the NF-κB and MAPK signaling pathways.

### 3.6. Cheonggukjang Improves Intestinal Epithelial Barrier Integrity by Modulating Mucins and Tight Junction Proteins in DSS-Induced Mice

**Figure 6:**
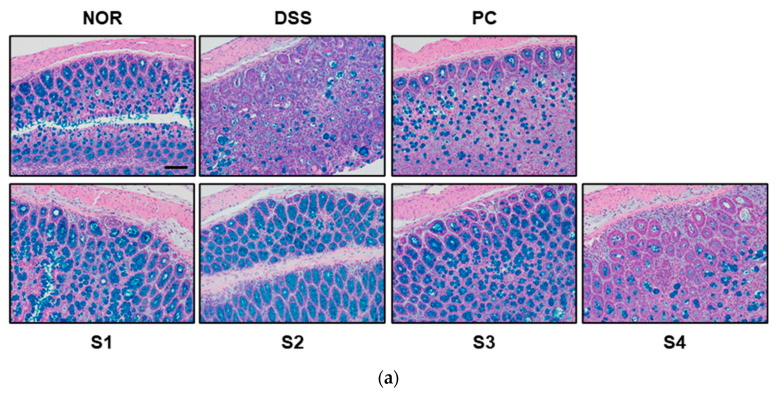

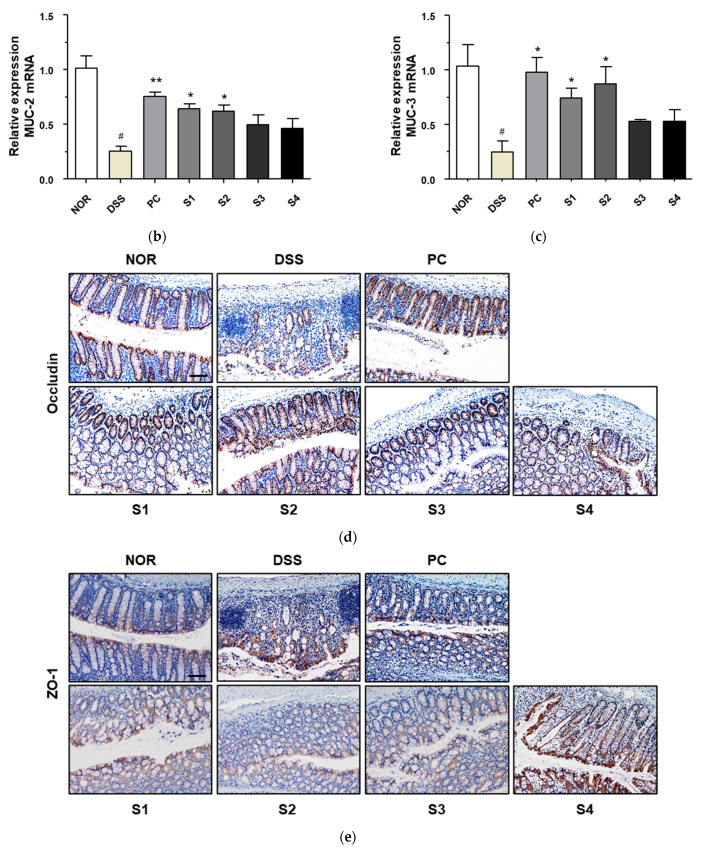

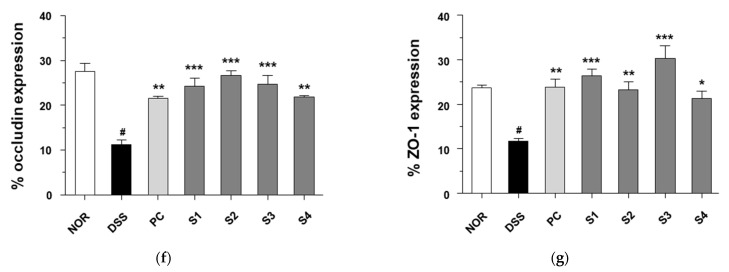
Effect of *Cheonggukjang* on the expression of mucins and tight junction protein in DSS-induced colitis mice: (**a**) Representative Alcian blue staining images of colon tissue. The abundance of goblet cells and mucin production was determined. Magnification (200×), Scale bar; 50 μm; mRNA levels of (**b**) *Muc2* and (**c**) *Muc3* were analyzed by quantitative real-time PCR; (**d**,**e**) Representative immunohistochemistry images of colon tissue. Magnification (200×), Scale bar, 50 μm; (**f**,**g**) Quantification of occludin and ZO-1 expression by IHC. Values are presented as the mean ± SD (n = 5); #, *p* < 0.005 versus normal group; ***, *p* < 0.001; **, *p* < 0.005; *, *p* < 0.05 versus DSS group.

In the normal intestine, mucins are expressed and secreted by goblet cells to protect the mucus layer; however, IBD often causes a loss of the mucin layer and goblet cell mucin [30]. Therefore, to evaluate histological changes, such as mucin and goblet cell depletion, Alcian blue staining was performed. As shown in Figure 6a, the control group contained well-organized mature goblet cells. However, the DSS-treated group showed histological changes, such as mucin and goblet cell depletion, compared to the control group. These histological changes were attenuated in the PC- and *Cheonggukjang* (S1–S4)-treated groups. Muc2 is a secreted gel-forming mucin and the main structural component of the protective mucus layer of the intestine [31]. It has been reported that mucin expression, such as Muc2 and Muc3, reduces with the progression of DSS-induced colitis in various animal model [30]. Therefore, we evaluated the expression of mucins to protect the mucus layer. As shown in Figure 6b,c, the reduced mRNA levels of *Muc2* and *Muc3* by DSS were attenuated upon treatment with PC and *Cheonggukjang* (S1–S4).

IBD compromises epithelial barrier functions by reducing tight junction proteins, including transmembrane barrier proteins (occludin) and cytoplasmic scaffolding proteins (ZO-1) [32]. Therefore, to examine the effect of *Cheonggukjang* samples (S1–S4) on intestinal barrier function after DSS treatment, tight junction protein expression was determined by immunohistochemical staining. As shown in Figure 6d,e, the expression levels of occludin and ZO-1 were significantly reduced in DSS-treated mice relative to those in the control group. However, PC and *Cheonggukjang* (S1–S4) treatment elevated the protein expression of occludin and ZO-1 compared to the DSS alone group. These results suggest that *Cheonggukjang* may improve intestinal epithelial barrier integrity by modulating mucins and tight junction proteins.

## 4. Conclusions

In this study, we analyzed the bacterial community structure of *Cheonggukjang* using traditionally made and commercial *Cheonggukjang* samples to evaluate whether the protective effect differs depending on the manufacturing method. We observed that both traditionally made and commercial *Cheonggukjang* contain various types of useful probiotics. We also observed that both traditional and commercial *Cheonggukjang* significantly ameliorated DSS-induced symptoms, such as body weight loss, colonic shortening, DAI, and histological changes, and improved intestinal epithelial barrier integrity on DSS-induced colitis mice. Furthermore, we showed that *Cheonggukjang* can attenuate DSS-induced colonic inflammation by suppression of NF-κB and MAPK signaling pathways. These findings suggest that *Cheonggukjang* can be useful as a functional food to improve inflammatory diseases such as colitis, regardless of the manufacturing method.

## Figures and Tables

**Table 1:** Disease activity index (DAI) score.

Score	Body Weight Decrease (%)	Stool Consistency	Fecal Bleeding
0	0	Normal	No bleeding
1	1–5		
2	5–10	Soft stools	Slight bleeding
3	11–15		
4	>15	Diarrhea	Gross bleeding

**Table 2:** Primer Sequences.

Gene	Forward (5′-3′)	Reverse (5′-3′)
*TNF-α*	AACTAGTGGTGCCAGCCGAT	CTTCACAGAGCAATGACTCC
*IL-6*	TGTCTATACCACTTCACAAGTCGGAG	GCACAACTCTTTTCTCATTTCCAC
*IL-1β*	GCAACTGTTCCTGAACTCAACT	ATCTTTTGGGGTCCGTCAACT
*iNOS*	CGAAACGCTTCACTTCCAA	TGAGCCTATATTGCTGTGGCT
*COX-2*	TTTGGTCTGGTGCCTGGTC	CTGCTGGTTTGGAATAGTTGCTC
*MUC-2*	GCAGTCCTCAGTGGCACCTC	CACCGTGGGGCTACTGGAGAG
*MUC-3*	CGTGGTCAACTGCGAGAATGG	CGGCTCTATCTCTACGCTCTC
*β-actin*	CGGTTCCGATGCCCTGAGGCTCTT	CGTCACACTTCATGATGGAATTGA

## Data Availability

Not applicable.
